# TaqMan Assay for Swedish *Chlamydia* *trachomatis* Variant

**DOI:** 10.3201/eid1309.070263

**Published:** 2007-09

**Authors:** Arnold Catsburg, Laura van Dommelen, Vitaly Smelov, Henry J.C. de Vries, Alevtina Savitcheva, Marius Domeika, Björn Herrmann, Sander Ouburg, Christian J.P.A. Hoebe, Anders Nilsson, Paul H.M. Savelkoul, Servaas A. Morré

**Affiliations:** * VU University Medical Center, Amsterdam, the Netherlands; † Academic Hospital Maastricht, Maastricht, the Netherlands; ‡ D.O. Ott Research Institute of Obstetrics and Gynaecology, St. Petersburg, Russia; § St. Petersburg State University, St. Petersburg, Russia; ¶ Health Service Amsterdam, Amsterdam, the Netherlands; # University of Amsterdam, Amsterdam, the Netherlands; ** Uppsala University, Uppsala, Sweden; †† Uppsala University Hospital, Uppsala, Sweden; ‡‡ South Limburg Public Health Service, Heerlen, the Netherlands; §§ City of Hope and Beckman Research Institute, Duarte, California, USA; 1 These authors contributed equally to the study.

**Keywords:** Chlamydia trachomatis, Swedish variant, diagnostics, real-time PCR, infectious diseases, outbreak, letter

**To the Editor:** *Chlamydia trachomatis* (CT) is the most prevalent bacterial sexually transmitted infection worldwide. Recently, a new variant of CT (swCT) has been reported in Halland County, Sweden. A total of 12 swCT specimens were sequenced and found to have the same deletion, a 377-bp deletion in the cryptic plasmid (*1*). Because the deletion was found in the target area of 2 commercial CT nucleic acid amplification tests (Roche, Basel, Switzerland, and Abbott Laboratories, Abbott Park, IL, USA), screening tests have produced false-negative results for patients infected with this new Swedish variant (*1*). In specific regions of Sweden, the proportion of all detected CT cases attributable to swCT ranges from 13% to 39%; a considerable number of chlamydia infections have escaped detection by commonly used test systems (*1*).

Although the first 2 studies to monitor potential spread of the swCT variant outside Sweden (Ireland and the Netherlands) did not detect swCT, a third study (Norway) did identify this variant (*2*–*4*). Subsequently, the European Surveillance of Sexually Transmitted Infections network and the European Center for Disease Prevention and Control launched an initiative, consisting of a short questionnaire, to learn more about this swCT variant problem outside Sweden (*5*).

However, quick monitoring of the spread of the swCT variant has been hampered by lack of a direct test to detect this swCT variant and by lack of a readily available positive control. We therefore constructed a positive control by using a clinical specimen of the swCT variant in which the deletion was present (forward swCT 5′-TCC GGA TAG TGA ATT ATA GAG ACT ATT TAA TC-3′ reverse swCT 5′GGT GTT TGT ACT AGA GGA CTT ACC TCT TC-3′) (*2*). The specimen was obtained in Sweden (by B.H.) and confirmed as swCT by the method described by Ripa and Nilsson (*6*). The obtained 98-bp amplicon was subsequently cloned in a pGEM-T Easy Vector (Promega Benelux b.v., Leiden, the Netherlands) and transformed in *Escherichia coli* DH5α. After extraction the plasmid was verified for the correct insert by sequencing and quantified as described (*7*). This positive control is available for researchers and clinicians free of charge.

Subsequently, we developed a real-time PCR (TaqMan assay) that specifically detects the swCt variant by using a probe that spans the 377-bp left and right gap border sequences: probe- swCT 5′-*^FAM^* GGA TCC GTT TGT TCT GG *^MGB^* -3′. One copy of cloned positive swCT control could be detected in our swCT assay. We selected 10 copies per PCR as positive swCT control for each run. A total of 239 recent samples known to be CT positive and identified with techniques detecting the swCT variant were retrospectively analyzed with our new swCT real-time PCR for 3 cohorts: 1) 30 real-time PCR CT-positive clinical samples (CT prevalence in the population, 1.8%) from the Department of Medical Microbiology and Infection Prevention, VU University Medical Center, Amsterdam, the Netherlands; 2) 57 Becton Dickinson (Franklin Lakes, NJ, USA) CT-positive samples (CT prevalence in the sexually transmitted disease population, 7.3%) from the Department of Infectious Diseases, South Limburg Public Health Service, Heerlen, the Netherlands; and 3) 152 CT-positive culture samples (CT prevalence in the population, average 15% [*8*]) from the Faculty of Medicine, St. Petersburg State University, St. Petersburg, Russia, and from the Laboratory of Microbiology, D.O. Ott Research Institute of Obstetrics and Gynaecology, St. Petersburg, Russia.

Cohort 1 consisted of cervical swabs in 2-sucrose-phosphate (2SP) transport medium, stored at –80°C. Cohort 2 consisted of frozen dry swabs that had been shaken for 10 s in 1 mL 2SP transport medium before sample preparation. Cohort 3 consisted of positive cultured samples. DNA extraction used 200 μL 2SP and was performed with the NucliSens easyMAG (bioMérieux, Boxtel, the Netherlands); the DNA was eluted in 110 μL 2SP (*7*). Presence of CT DNA was reconfirmed for all samples with our in-house PCR. Sensitivity of this assay was determined by using a previously described serial dilution of lymphogranuloma venereum (LGV) strain L2 and was assessed at 0.01 inclusion-forming units (*9*). Amplification and detection were performed with an ABI Prism 7000 sequence detection system (Applied Biosystems, Foster City, CA, USA) by standard PCR conditions of the manufacturer, with 45 cycles. The Swedish variant was found in none of the 3 cohorts tested. Sensitivity and specificity were confirmed by using 12 swCT variant samples from Sweden, which were all positive according to our swCT TaqMan assay.

Our new swCT TaqMan assay, combined with the positive control (which can be obtained by contacting S.M.), will be a helpful tool for determining whether this Swedish CT variant is present outside Sweden, other than in the 2 case-patients identified in Norway. We did not find any evidence of the swCT variant in the Netherlands or St. Petersburg, Russia, each of which is near Scandinavia (Table). Recently, the *C. trachomatis* LGV strain was discovered in the Netherlands in a population of men who have sex with men. In this instance, the real-time TaqMan assay also proved helpful in determining spread (*10*).

**Table:** Published studies and the current study on screening for the swCT variant*

Location	Ct+, no. detected	swCT variant, no. detected	Reference
Amsterdam, the Netherlands	75	ND	(*3*)
Dublin, Ireland	750	ND	(*4*)
Oslo, Norway†	47	2	(*5*)
St. Petersburg, Russia	152	ND	This study
Heerlen, the Netherlands	57	ND	This study
Amsterdam, the Netherlands	30	ND	This study

*swCT, Swedish *Chlamydia trachomatis* variant identified in Halland County, Sweden; Ct+, *C. trachomatis* DNA; ND, not detected. †2 female patients: 1 originally from Sweden, 1 from Norway.
